# Permeability of Fresh and Frozen Porcine and Human Gingiva and the Effect of Storage Duration

**DOI:** 10.3390/pharmaceutics15051492

**Published:** 2023-05-13

**Authors:** Apipa Wanasathop, Hyojin Alex Choi, Patcharawan Nimmansophon, Michael Murawsky, Deepak G. Krishnan, S. Kevin Li

**Affiliations:** 1 Division of Pharmaceutical Sciences, College of Pharmacy, University of Cincinnati, Cincinnati, OH 45267, USA; 2 Division of Oral and Maxillofacial Surgery, College of Medicine, University of Cincinnati, Cincinnati, OH 45267, USA

**Keywords:** porcine gingiva, permeability, diffusion cell, oral mucosa, transport

## Abstract

The gingiva is the target site for some topical drugs, but the permeability of human gingiva has not been systematically evaluated. Pigs are a common animal model for in vitro membrane transport studies. The objectives of this study were to: (a) determine the permeability coefficients of freshly excised human gingiva using model permeants, (b) compare the permeability coefficients of fresh human gingiva with those of fresh porcine gingiva, (c) evaluate the effect of freezing duration on the permeability of porcine gingiva, and (d) compare the permeability coefficients of fresh and cadaver (frozen) human gingiva. A goal was to examine the feasibility of using porcine gingiva as a surrogate for human gingiva. The potential of using frozen tissues in permeability studies of gingiva was also examined. Fresh and frozen porcine gingiva, fresh human gingiva, and frozen cadaver human gingiva were compared in the transport study with model polar and lipophilic permeants. The fresh porcine and human tissues showed similarities in the “permeability coefficient vs. octanol–water distribution coefficient” relationship. The porcine gingiva had a lower permeability than that of the human, with a moderate correlation between the permeability of the fresh porcine and fresh human tissues. The permeability of the porcine tissues for the model polar permeants increased significantly after the tissues were frozen in storage. Moreover, the frozen human cadaver tissue could not be utilized due to the high and indiscriminating permeability of the tissue for the permeants and large tissue sample-to-sample variabilities.

## 1. Introduction

Animal tissues are routinely used as an alternative to human tissues in drug permeation studies. The oral mucosae of pigs, dogs, rabbits, and rhesus monkeys are known to be similar to those of humans [1,2,3]. Pigs are a common animal model used in in vitro drug transport studies of oral mucosae. Pig tissues have the advantage of being abundant and able to be obtained in large quantities. A porcine-derived acellular dermal matrix was also used in soft tissue replacement grafts to treat gingival recession defects [4]. In vitro or ex vivo experiments utilizing animal tissues offer considerable advantages. However, one of the difficulties is the proper storage of the excised tissue specimens [5]. The need for tissue storage is due to the limited availability of fresh tissues. Tissue storage conditions can vary. Several storage methods have been used for oral mucosa and these methods have shown that freezing does not significantly affect the permeability of the tissue [6,7,8]. On the contrary, some studies have shown that freezing could impact the permeability of the tissue [5,9], as ice crystal formation can cause cell damage due to cellular stress and deformation during freezing [10].

The gingiva is a part of the periodontium, which provides an effective barrier to both mechanical trauma and bacterial invasion [11,12,13,14]. Understanding the transport barrier of the gingiva could provide insights into effective topical drug delivery for the gingiva and its surrounding tissues, such as those related to periodontal diseases. A recent study investigated the permeability of the gingiva using 12 model permeants and a modified Franz diffusion cell, with porcine tissue as the human tissue surrogate in vitro [15]. For the porcine gingiva, the lipophilic permeants exhibited higher permeability coefficients than the hydrophilic permeants and the molecular weight of the permeants affected the permeability. In addition, a three-factor correlation was observed between the logarithm of the permeability coefficient (Log *kp*), logarithm of the octanol–water distribution coefficient (Log *D_ow_*), and molecular weight (*MW*) of the permeants. This correlation also suggested that the gingiva barrier was less lipophilic than octanol [15].

The objectives of the present study were to: (a) determine the permeability coefficients of freshly excised human gingiva, (b) compare the permeability coefficients of fresh human gingiva with those of fresh porcine gingiva, (c) evaluate the effect of freezing duration on the permeability of porcine gingiva, and (d) compare the permeability coefficients of fresh and cadaver (frozen) human gingiva. The goal was to assess the feasibility of using porcine gingiva as a surrogate for human tissue in transport studies on drug delivery. The potential of using frozen tissues in permeability studies of gingiva was also examined. Fresh and frozen porcine gingiva, fresh human gingiva, and frozen cadaver human gingiva were used in the investigation. The transport studies using model permeants with different lipophilicities were performed in a modified Franz diffusion cell. The fresh and frozen porcine and human gingiva permeability coefficients were compared, as well as the impacts on the tissue barrier integrity from different storage conditions. The approach was to systematically study the porcine tissues under different conditions (fresh and frozen for 1–4 weeks) and then compare the results using the human tissues (fresh and frozen cadavers).

## 2. Materials and Methods

### 2.1. Materials

^14^C-Urea, ^3^H-estradiol, and ^3^H-fluconazole were purchased from Moravek Biomaterials and Radiochemicals (Brea, CA, USA). ^14^C-Tetraethylammonium bromide, ^14^C-salicylic acid, ^3^H-mannitol, ^14^C-sucrose, and Ultima Gold scintillation cocktail were from PerkinElmer Life and Analytical Sciences (Boston, MA, USA). ^14^C-Indomethacin was from Amersham Life Science (Amersham, United Kingdom). ^3^H-Corticosterone was from American Radiolabeled Chemicals (St. Louis, MO, USA). Chlorhexidine diglucanote solution (20% in water), sucrose, and sodium salicylate were purchased from Sigma–Aldrich (St. Louis, MO, USA). Tetraethylammonium bromide, urea, mannitol, corticosterone, indomethacin, and sodium azide (NaN_3_) were purchased from Acros Organics (Morris Plains, NJ, USA). Ketorolac tromethamine and estradiol were from Letco Medical (Decatur, AL, USA). Fluconazole was from TCI America (Portland, OR, USA). Lidocaine HCl was from the Professional Compounding Center of America (PCCA, Houston, TX, USA). Triethylamine (HPLC grade), monobasic sodium phosphate, and glacial acetic acid were purchased from Fisher Scientific (Fair Lawn, NJ, USA). Acetonitrile (HPLC grade) was from Pharmaco-AAPER (Shelbyville, KY, USA). Phosphate-buffered saline (PBS), pH 7.4, consisting of 0.01 M phosphate buffer, 0.0027 M potassium chloride, and 0.137 M sodium chloride was prepared using PBS tablets (MP Biomedicals, LLC, Solon, OH, USA) and deionized water (DI water). All the materials were used as received.

### 2.2. Preparation of Fresh and Frozen Porcine Tissue

The fresh porcine gingiva tissues were obtained from female mix Yorkshire pigs (70–100 days old, Isler Genetics, Prospect, OH, USA). More than 60 samples were collected for the studies in the gingival drug delivery project (n = 19 pigs). These tissues were collected from animal carcasses donated by other unrelated animal studies in the Laboratory Animal Medical Services (LAMS) facility at the University of Cincinnati (Cincinnati, OH, USA). They were donated post-sacrifice and used immediately. Within an hour after the animals were sacrificed, a scalpel was used to cut around selected areas of the gingiva and the tissues were removed through blunt dissection. After the tissues were harvested, they were immersed in a 0.9% normal saline solution. The excess connective tissues were removed and the tissue thicknesses were measured by a micrometer (Mitutoyo Corporation, Japan). For the studies with fresh porcine tissues, the tissues were used immediately or after a 4 °C refrigeration, where they were mounted on the modified Franz diffusion cells, as stated in Section 2.4 and Section 2.5. Table 1 summarizes the storage conditions (temperature and duration) of the tissues. For the studies with the frozen porcine tissues, after the excess connective tissues were removed, the excess liquid on the gingiva tissues was removed by lightly pressing the tissues with Kimwipe paper. The tissues were then immediately wrapped in aluminum foil and stored at −20 °C until use. These tissues were stored for either 1, 2, or 4 weeks (see Table 1) for the transport study in Section 2.5. In the transport study, the frozen tissues were allowed to thaw in PBS with 0.02% sodium azide for 15 min before being mounted on the modified Franz diffusion cells. To mount the tissues on the diffusion cells, each tissue was sandwiched between the glass slides of the diffusion cell chambers. In total, 0.5 and 5 mL of PBS with 0.02% sodium azide were added to the donor and receptor chambers, respectively, before the start of the experiment on the same day.

### 2.3. Preparation of Fresh and Frozen Human Tissue

The fresh human gingiva tissues were obtained from patients (both male and female, aged between 11 and 76 years old) who had full-mouth tooth extractions in an outpatient clinic of the Department of Surgery, Division of Oral and Maxillofacial Surgery, University of Cincinnati (Cincinnati, OH, USA). The tissues were discarded as a part of the tooth extraction procedures and deidentified. More than 50 samples of the discarded tissues were obtained for the studies in the gingival drug delivery project (n = 25 human donors). These tissues were mostly attached gingiva near the teeth from different locations in the oral cavity. Only the tissues normal in appearance via visual inspection were collected. The tissues were immediately stored in PBS and used on the same day for the electrical resistance and transport studies in Section 2.4 and Section 2.5 (see summary in Table 1). The frozen human cadaver gingiva tissues (frozen fresh) were obtained from the mandibles (n = 3 human donors, aged between 47 and 71 years old) provided by Science Care (Phoenix, AZ, USA). The selection criteria included: (a) a good general condition via visual inspection, (b) no known periodontal diseases, (c) a non-heavy smoker status, and (d) non-diabetics. The mandible tissue was procured within 4–8 days after death and shipped within 2–4 days. The mandible was stored at −20 °C and used within 2 days after the receipt of the tissue. To obtain the gingiva samples, the mandible was allowed to thaw in PBS for 1 h before dissection. The top part of the gingiva (attached gingiva), close to the teeth, which contained the keratinized layers, was selected and cut into approximately 0.3 cm × 0.3 cm gingiva samples using a scalpel and blunt dissection. For both the fresh and frozen human tissues, the excess connective tissues were removed and the tissue thicknesses were measured using a micrometer. Before the studies in Section 2.4 and Section 2.5, the tissues were mounted on the modified Franz diffusion cells. Super glue was applied around the edge of each tissue to hold it in place and then the tissue was sandwiched between the glass slides of the diffusion cell chambers. In total, 0.5 and 5 mL of PBS with 0.02% sodium azide were added to the donor and receptor chambers, respectively, before the start of the experiment. The use of deidentified human tissue specimens was approved by the Institutional Review Board (IRB) at the University of Cincinnati (Cincinnati, OH, USA), IRB #2019-0959.

### 2.4. Electrical Resistance Measurement

The stability of the tissue barrier was evaluated using the tissue electrical resistance in the stability study. The tissue electrical resistance was monitored in the Franz diffusion cell, as described in a previous study [15]. Briefly, the tissue resistance was measured using Ohm’s law and an electrical system with a 1.5 V battery, two voltmeters (Fluke Model 73III or Model 177; Everett, WA, USA), and a 375-kΩ fixed resistor coupled in series with Ag and Ag/AgCl electrodes. The voltage applied across the tissue was in the range of 0.03–0.50 V, depending on the tissue resistance.

The initial resistance of the tissue was measured at room temperature immediately after the tissue collection and before and after storage under the specified conditions. For the measurement after storage at 4 °C, the tissue was allowed to equilibrate at room temperature for 1 h before the measurement. For the measurement when the tissue was at 34 °C, the tissue was maintained and measured at 34 °C over the duration of the experiment.

### 2.5. Transport Study for Frozen Porcine and Human Gingiva

The transport study on the gingiva was performed as described in the previous study [15]. Briefly, after the tissue was mounted on the modified Franz diffusion cell (with a diffusional area of 0.03 cm^2^ by using two glass slides, each with a drilled hole of 0.2-cm in diameter), micro-magnetic stir bars were placed in both the donor and receptor chambers to provide stirring. The diffusion cell was placed on a 38 °C thermostated heating and stirring module. The resulting temperature of the tissue was 34 °C during the experiment. The temperature was monitored using an IR thermal camera (FLIR-E63900, FLIR Systems, Täby, Sweden). The tissue was allowed to equilibrate in the diffusion cell for 1 h before the start of the transport experiment. The conditions of the experiment and the physicochemical properties of the model permeants are listed in Table 2. Due to the limited availability of human tissues, some permeants used in the porcine studies (that have similar physicochemical properties) were not selected for the human studies. To start the experiment, the PBS in the donor chamber was replaced with a 0.5 mL donor solution. The donor chamber was then sealed with parafilm to prevent evaporation during the experiment. The sampling timepoints were 4, 8, 12, 16, and 24 h for the porcine gingiva and 2, 3, 4, 5, and 6 h for the human gingiva, respectively. At these timepoints, a 1 mL receptor solution and 10 µL donor solution were taken from the diffusion cell chambers and assayed for the permeants. After that, 1 mL of fresh PBS was added to the receptor to maintain a constant volume in the receptor.

### 2.6. Assay

For the radiolabeled permeants, urea, mannitol, sucrose, tetraethylammonium (TEA), salicylic acid, corticosterone, estradiol, indomethacin, and fluconazole, the samples were mixed with a 5-mL scintillation cocktail and analyzed with a liquid scintillation counter (Beckman Coulter LS6500, Fullerton, CA, USA). For the permeants of lidocaine, ketorolac, and chlorhexidine, the samples were assayed using HPLC (Prominence; Shimadzu, Columbia, MD, USA) with a C8 column at room temperature. The mobile phase for lidocaine was 76% DI water, 20% acetonitrile, and 4% glacial acetic acid at a pH of 3.4. The mobile phase for ketorolac and chlorhexidine was 70% DI water and 30% acetonitrile with 0.12 M monobasic sodium phosphate and 0.5% triethylamine, at a pH of 3.0. The flow rate was 1.5 mL/min. The detection wavelengths were 254, 313, and 239 nm for lidocaine, ketorolac, and chlorhexidine, respectively.

### 2.7. Data Analysis

The data obtained in the present study are presented as means and standard deviations (SD). All the experiments were performed at least in triplicate. The statistical analyses were performed using one-way ANOVA with GraphPad (La Jolla, CA, USA) and *F*-tests on the variance with Microsoft Excel (Redmond, WA, USA), and a difference of *p* < 0.05 was considered as statistically significant.

## 3. Results and Discussion

### 3.1. Comparison of Electrical Resistance of Gingiva

**Figure 1:**
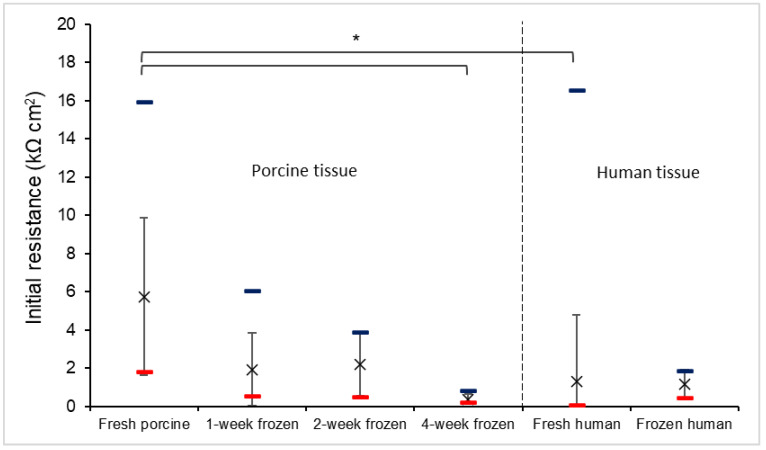
Initial electrical resistance of each type of tissues. Mean ± SD and minimum and maximum (lower and upper dashes, respectively). The mean and SD were calculated by treating the result of each donor as a single data point. The resistance of each donor was the average value of the resistances of the samples from the same donor. Sample size: 70 total samples from n = 19 tissue donors for fresh porcine, 12 samples from n = 8 donors for 1-week frozen porcine, 7 samples from n = 4 donors for 2-week frozen porcine, 12 samples from n = 6 donors for 4-week frozen porcine, 58 samples from n = 25 donors for fresh human, and 67 samples from n = 3 donors for frozen human tissues, respectively. The asterisk indicates significant differences between the resistance of the fresh and 4-week frozen porcine tissues and between the resistance of fresh porcine and human tissues (one-way ANOVA, * *p* < 0.05).

The initial electrical resistances of the gingiva tissues with the different storage conditions are presented in Figure 1. The initial resistances were measured as described in Section 2.4. For the porcine tissues, the electrical resistance decreased with the storage duration (fresh vs. 4-week frozen tissues, one-way ANOVA, *p* < 0.05). When the tissues were frozen, the freezing of the water in the tissues might have altered the tissue barriers. In addition, a large electrical resistance range was observed for the porcine tissues. Similar to the porcine tissues, a large electrical resistance range was also observed among the fresh human tissues. However, unlike the fresh porcine tissues, in which the intra-subject variability (sample-to-sample variability) and inter-subject variability (donor-to-donor variability) were not significantly different (*F*-test, *p* = 0.95), the fresh human tissues exhibited a large inter-subject variability (*F*-test, *p* < 0.05). Some fresh human tissues had high resistance values, but the mean values of all fresh human tissues were lower than those of the fresh porcine tissues (fresh human vs. fresh porcine tissues, one-way ANOVA, *p* < 0.05). This large variability might be due to several factors: (a) the inherited variability from the different donors could be significant for the human tissues, (b) the fresh human tissues were obtained from patients and some of these tissues might have had pathological conditions, in that their barrier functions were different from those in healthy human tissues, and (c) the human fresh tissues required extra handling (e.g., the tissue transfer from the clinic to the lab) and this process could have led to tissue damage. The frozen human tissues showed a narrower resistance range, with the mean value being close to that of the fresh human tissues.

The difference between the electrical resistances of the fresh porcine and human tissues could be related to the thicknesses of their epithelial layers. To compare the thicknesses of the epithelium of the fresh porcine and human tissues, the thicknesses were measured with microscopy, using slides prepared by embedding the tissues in paraffin and hematoxylin and eosin (H&E) staining. The thicknesses of the keratinized and epithelium layers were 37 ± 7 µm and 402 ± 45 µm for the fresh porcine tissue, and 14 ± 14 µm and 359 ± 67 µm for the fresh human tissue, respectively (Mean ± SD, n = 4). The ranges of the epithelium layer thicknesses of the porcine tissues were 332–519 µm (mean ± SD = 402 ± 45 µm), 206–492 µm (mean ± SD = 345 ± 120 µm), 162–377 µm (mean ± SD = 246 ± 66 µm), and 187–534 µm (mean ± SD = 360 ± 159 µm) for the fresh, 1-week, 2-week, and 4-week storage conditions. These values were within the same range as those reported previously (35 µm keratinized layer and 208 µm epithelium for porcine [16] and 285 µm epithelium for human [17]).

**Figure 2:**
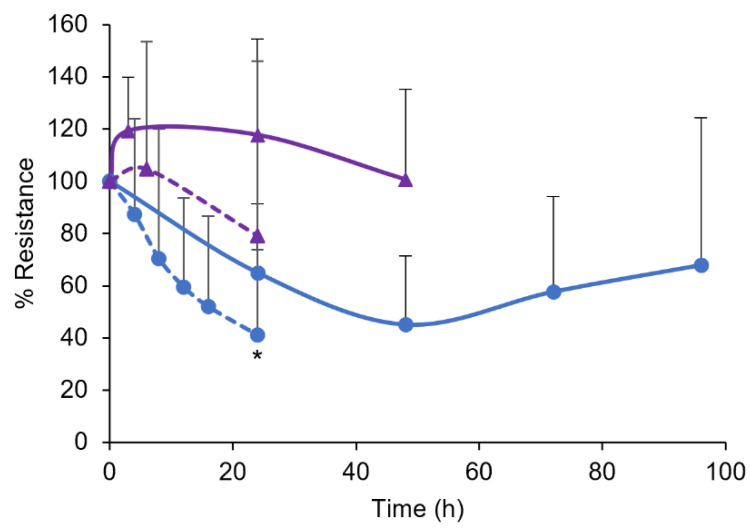
Changes in electrical resistance of fresh porcine (blue circles) and fresh human (purple triangles) gingiva over time at 4 °C (solid line) and 34 °C (dashed line) storage temperatures. Mean ± SD, n = 3–8. The asterisk indicates significant differences between the 0 and 24 h data (one-way ANOVA, * *p* < 0.05).

The tissue stability was evaluated by monitoring the electrical resistances of the fresh porcine and human tissues. Figure 2 compares the changes in the electrical resistance (as % initial resistance) of the fresh porcine gingiva and fresh human gingiva over time at different storage temperatures, 4 °C and 34 °C. Both tissues were relatively stable for at least 2 days when stored at 4 °C. The resistance of the porcine tissues fluctuated within the variability over time but did not show any significant difference at 144 h (one-way ANOVA, *p* > 0.05). Regardless, the fresh human tissues were used within the same day of extraction in the transport study (Section 3.3) to minimize possible tissue degradation. When the tissues were equilibrated at the 34 °C experimental temperature, the average electrical resistance of the tissues began to decrease, with a significant difference for the porcine tissues at 24 h. For these samples, the average electrical resistances of the human and porcine tissues decreased to approximately 80% and 42% after 24 h at 34 °C. Therefore, the 24 h timepoint was selected to be the last sampling time in the transport study for the porcine tissues.

### 3.2. Effect of Storage Duration on the Permeability of Porcine Gingiva

**Figure 3:**
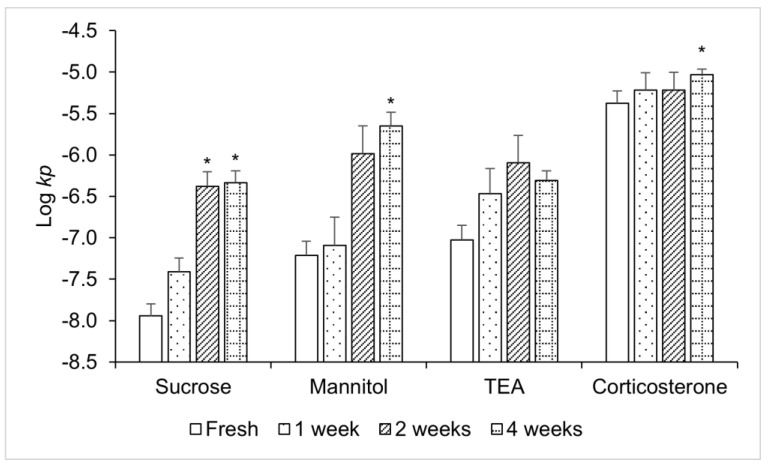
Effect of storage duration at −20 °C on the logarithm of permeability coefficient (Log *kp*) of porcine gingiva. Mean ± SD, n = 4–6 for each permeant. The asterisks indicate significant differences between the permeability coefficients of the fresh and frozen tissues (one-way ANOVA, * *p* < 0.05).

The Log *kp* results of the tissues stored over different durations for the model permeants are shown in Figure 3. The frozen porcine tissues were stored at −20 °C for either 1, 2, or 4 weeks before the transport experiment. The permeability coefficients of most of the model permeants increased significantly (for sucrose, mannitol, and corticosterone; one-way ANOVA, *p* < 0.05) when the tissues were stored for 4 weeks. For polar permeants such as sucrose, the permeability coefficients of the gingiva stored for 2 weeks were also significantly higher than those of the fresh gingiva (one-way ANOVA, *p <* 0.05). Corticosterone, a lipophilic permeant, displayed relatively little change in its permeability during the first 2 weeks of storage, suggesting that the damage to the porcine gingiva under this condition might not yet have a significant impact on the tissue barrier for the lipophilic permeants. Using a parallel transport pathway model to interpret the data, the permeant transport across the gingiva can be described by the transport across the lipoidal pathway (transcellular transport across the membrane lipid barrier) and polar pathway (paracellular transport across aqueous channels or transport via membrane defects) [18,19]. Based on the lipophilicity of corticosterone (measured by its octanol/water partition coefficient (*K_ow_*); Log *K_ow_* = 1.9), it is expected to penetrate the tissue mainly via the lipoidal pathway, whereas water-soluble compounds such as sucrose and mannitol are expected to predominately penetrate via the polar pathway. The result could indicate that the polar pathway was more prone to the effect of freezing than the lipoidal pathway. The formation of the ice crystal in the polar pathway of the tissue under the storage conditions might contribute to this effect.

Similar results were observed with buccal tissue in previous studies. For example, the permeability of ^3^H-water was not altered when porcine buccal tissue was stored, intact, at 4 °C in a pig head. However, the permeability increased significantly when the buccal tissue was frozen at −20 °C for 24 h [5]. The permeability of lidocaine hydrochloride significantly increased for the dorsum of the tongue and buccal epithelia after four weeks of storage at −20 °C [20]. The steady state fluxes for water, arecoline, and vasopressin were significantly different in fresh and frozen/thawed porcine buccal mucosa [9]. Canine buccal mucosa was interestingly not affected by storage for up to five weeks when stored in a balanced salt solution at 4 °C [21]. In the present study, frozen human gingiva tissues were investigated and compared to the porcine data (see Section 3.3).

### 3.3. Transport Study of Fresh and Frozen Human Gingiva

**Figure 4:**
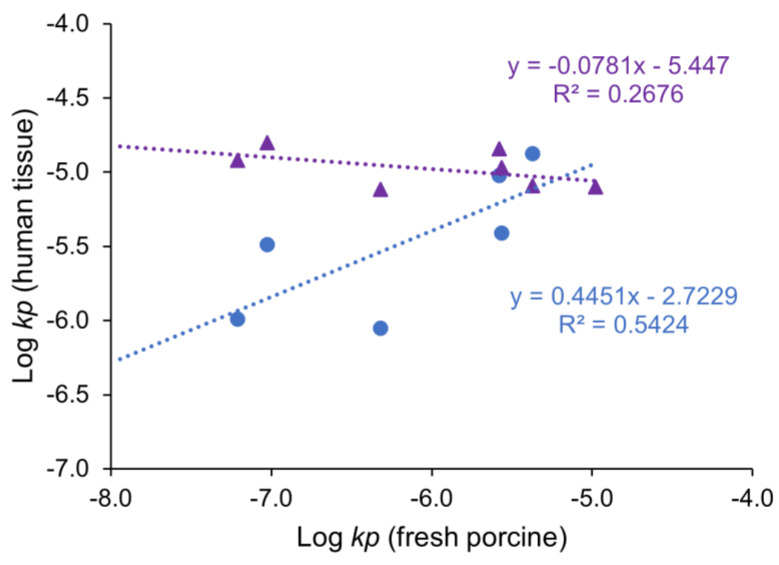
Correlation between the logarithm of permeability coefficient (Log *kp*) of fresh porcine gingiva and fresh human (circles) or frozen human (triangles) gingiva. Mean ± SD, n = 4–6 for each permeant. Each data point represents the permeability coefficient of a single permeant obtained from the tissue.

The correlation between the Log *kp* of fresh human gingiva and porcine gingiva tissues is shown in Figure 4. There was no direct correlation between the frozen human gingiva and fresh porcine gingiva tissues (triangles in the figure). Different from the frozen human gingiva, a better correlation was observed between the fresh human and porcine gingiva tissues (circles in the figure), albeit a correlation that was only moderate. For the model permeants, the fresh human gingiva consistently showed higher permeability coefficients than those of the fresh porcine gingiva; the permeability coefficients of the fresh human gingiva were approximately 1-4x higher than those of the fresh porcine gingiva, except for the polar permeant mannitol and ionic permeant tetraethylammonium. Lesch et al. also observed that the floor of the human mouth tissue was more permeable than that of the pig, but no significant differences were found in the buccal tissues when using ^3^H-water [6]. In the present study, despite the difference in permeability between the human and porcine tissues, both fresh tissues showed similar trends of tissue permeability vs. permeant lipophilicity relationships for the lipophilic and polar permeants. This suggests that the fresh porcine gingiva tissue (but not the frozen human tissue) could provide insights into human gingival drug delivery and be utilized as a surrogate for fresh human gingiva.

**Figure 5:**
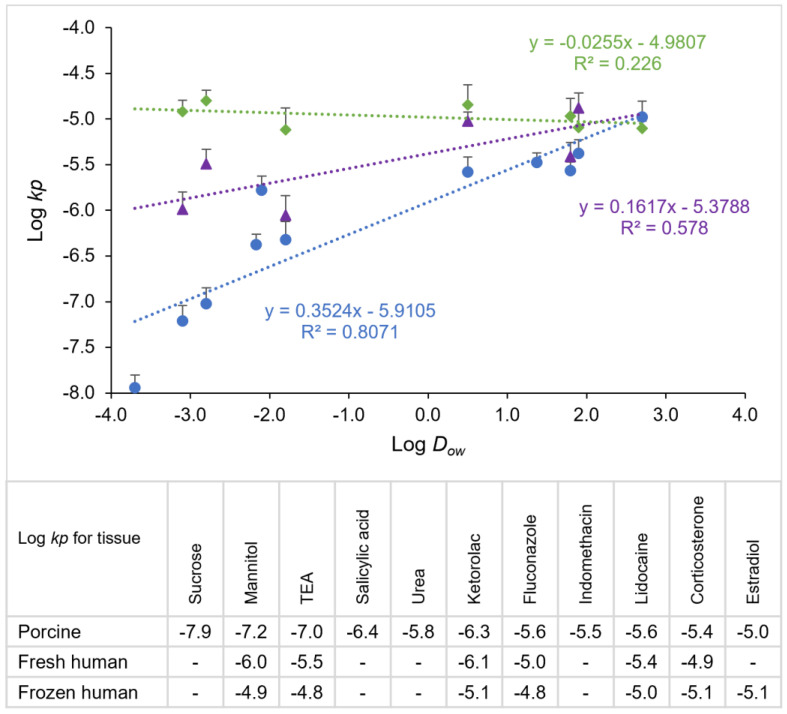
Relationship between the logarithm of permeability coefficient (Log *kp*) and logarithm of octanol–water distribution coefficient (Log *D_ow_*) for fresh porcine (blue circles), fresh human (purple triangles), and frozen human (green diamonds) gingiva. Mean ± SD, n = 4–6 for each permeant. Each data point represents the permeability coefficient of a single permeant obtained from the tissue.

Figure 5 shows the relationships between the Log *kp* and Log octanol–water distribution coefficients (Log *D_ow_*) of the permeants. The permeability coefficients increased with the distribution coefficients of the permeants, with least squares linear regression slopes of 0.35 and 0.16 and y-intercepts of −5.91 and −5.38 for the fresh porcine and fresh human gingiva, respectively. The differences between these tissues could be attributed to (a) the difference in the thicknesses of the epithelial layers in the tissues (see Section 3.1), (b) the conditions of the tissues (e.g., healthy vs. inflammatory states), and (c) the other intrinsic properties of the tissues (e.g., the lipophilicity and fluidity of the lipids in the barrier). The conditions of the tissues from the patients could also affect the tissue barrier function, as the fresh human gingiva tissues were not necessarily obtained from healthy donors. For example, pathological conditions (e.g., tissue inflammation) might not be visible during the tissue procurement in the full-mouth tooth extraction procedure. Further investigations of fresh, healthy human gingiva without any pathological conditions are required to examine this hypothesis.

No relationship was observed between the Log *kp* and Log *D_ow_* for the frozen human tissues from cadavers. There was a large sample-to-sample variability and the permeability coefficients of the frozen tissues were relatively constant over the range of the permeants studied. In addition, the permeability coefficients of the frozen human tissues for the polar permeants were significantly larger than those of the fresh human and porcine tissues. This is consistent with the results of the frozen porcine tissues in Section 3.2, in that freezing the gingiva could significantly increase the permeability of the polar permeants. The permeability (barrier) of the human gingiva could be compromised by the storage (freezing) conditions. It is also possible that the barrier of the gingiva was damaged in the preparation of the cadaver tissues and/or affected by the conditions of the tissues from the human donors. The conditions of the epithelial layer of the gingiva, depending on the inflammatory state, can vary from one individual to another (human or pig) and distort the results. It should be noted that the frozen human tissues were obtained from the mandible only, whereas the other tissues were from the mandible and maxilla, and possible differences can exist between the gingiva from the mandible and maxilla. Future studies are required to investigate these questions.

## 4. Conclusions

The permeability coefficients of excised human gingiva obtained from patients were determined with six model permeants. The fresh human and porcine tissues showed similar Log *kp* vs. Log *D_ow_* relationships for the permeants, where the permeability coefficients increased with the octanol-water distribution coefficients of the permeants. There was a moderate correlation between the permeability coefficients of the fresh human gingiva and fresh porcine gingiva. The permeability of the fresh human gingiva was generally higher than that of the fresh porcine gingiva. With a difficulty in obtaining fresh tissue, frozen tissues can be used. Freezing the porcine gingiva did not significantly affect the permeability coefficients of a moderate lipophilic compound for 2 weeks, but the permeability coefficients of the polar compounds increased significantly after the tissues were frozen. No correlation was observed between the Log *kp* and Log *D_ow_* for the frozen human tissues from cadaver mandibles. In addition, there was no correlation between the permeability coefficients of the frozen and fresh human tissues for the polar permeants.

## Figures and Tables

**Table 1:** Tissue sources and storage conditions.

Tissue	Source and Study Condition	Storage Condition
Porcine	Fresh	4 °C, used within 2 days
	Frozen, 1-week storage	−20 °C, 1 week
	Frozen, 2-week storage	−20 °C, 2 weeks
	Frozen, 4-week storage	−20 °C, 4 weeks
Human	Fresh, discarded from a dental clinic	4 °C, used within the same day
	Frozen cadaver, within 2 weeks after date of death	Stored intact within the lower mandible, procured and frozen at −20 °C within 4–8 days, shipped within 2–4 days, and used within 2 days after receipt

**Table 2:** Physicochemical properties of the permeants, conditions of the transport study, and the donor concentrations used in the study.

Permeant	MW (g/mol)	Log *K_ow_*	pKa	Log *D_ow_* ^a^	Concentration Used (mg/mL)	Study Condition
Chlorhexidine	505	1.3	10.8	0.1	53.5	Fresh porcine and human
Corticosterone	347	1.9	--- ^b^	1.9	0.1	All tissue
Estradiol	272	4.0	--- ^b^	2.7	0.001	Fresh porcine and frozen human
Fluconazole	306	0.5	--- ^b^	0.5	0.001	All tissue
Indomethacin	358	4.3	4.5	1.4	0.001	Fresh porcine only
Ketorolac	255	2.1	3.5	−1.8	10	All tissue
Lidocaine	234	2.4	7.9	1.8	10	All tissue
Mannitol	182	−3.1	--- ^b^	−3.1	0.1	All tissue
Salicylic acid	138	2.3	3.0	−2.2	0.1	Fresh porcine only
Sucrose	342	−3.7	--- ^b^	−3.7	0.1	Fresh porcine only
TEA	130	−2.8	--- ^b^	−2.8	0.1	All tissue
Urea	60	−2.1	--- ^b^	−2.1	0.001	Fresh porcine only

^a^ The octanol/water distribution coefficient (*D_ow_*) was calculated by $D_{ow}=K_{ow}f_{u}$, where *K_ow_* is the octanol/water partition coefficient and $f_{u}$ is the fraction of unionized permeant calculated from the *pKa* at pH 7.4 ($f_{u}=1/\left(1+10^{\left(pH-pKa\right)}\right)$ for weak acid and $f_{u}=1/\left(1+10^{\left(pKa-pH\right)}\right)$ for weak base). ^b^ Not applicable.

## Data Availability

The data presented in this study are available on request from the corresponding author.
